# Piloting an automated query and scoring system to facilitate APDS patient identification from health systems

**DOI:** 10.3389/fimmu.2024.1508780

**Published:** 2025-01-21

**Authors:** Amy M. FitzPatrick, Aaron T. Chin, Sharon Nirenberg, Charlotte Cunningham-Rundles, Keith Sacco, Jesse Perlmutter, Joseph F. Dasso, Athanasios Tsalatsanis, Jay Maru, Jessica Creech, Jolan E. Walter, Nicholas Hartog, Neema Izadi, Mandy Palmucci, Manish J. Butte, Klaus Loewy, Anurag Relan, Nicholas L. Rider

**Affiliations:** ^1^ Precision AQ, Bethesda, MD, United States; ^2^ Department of Pediatrics, University of California, Los Angeles, Los Angeles, CA, United States; ^3^ Division of Informatics and Data Architecture, Icahn School of Medicine, Departments of Scientific Computing and Data, Mount Sinai School of Medicine, New York, NY, United States; ^4^ Division of Clinical Immunology, Icahn School of Medicine, Departments of Medicine and Pediatrics, Mount Sinai School of Medicine, New York, NY, United States; ^5^ Department of Child Health, University of Arizona College of Medicine and Division of Pulmonology, Section of Allergy-Immunology, Phoenix Children’s Hospital, Phoenix, AZ, United States; ^6^ Phoenix Children’s Hospital, Phoenix, AZ, United States; ^7^ Department of Pediatric Allergy and Immunology, University of South Florida at Johns Hopkins All Children’s Hospital, St. Petersburg, FL, United States; ^8^ Research Methodology and Biostatistics Core, Morsani College of Medicine, University of South Florida Health, St. Petersburg, FL, United States; ^9^ Management Analyst, Research Methodology and Biostatistics Core, Morsani College of Medicine, University of South Florida Health, St. Petersburg, FL, United States; ^10^ Department of Pediatrics, University of South Florida at Johns Hopkins All Children’s Hospital, St. Petersburg, FL, United States; ^11^ Division of Allergy and Immunology, Helen DeVos Children’s Hospital and Corewell Health, Grand Rapids, Michigan State University College of Human Medicine, East Lansing, MI, United States; ^12^ Division of Clinical Immunology and Allergy, Children’s Hospital Los Angeles, University of Southern California, Los Angeles, CA, United States; ^13^ Division of Information Services, Children’s Hospital Los Angeles, University of Southern California, Los Angeles, CA, United States; ^14^ Department of Pediatrics and Department of Microbiology, Immunology, and Molecular Genetics, University of California, Los Angeles, Los Angeles, CA, United States; ^15^ Department of Information Services, Texas Children’s Hospital, Houston, TX, United States; ^16^ Pharming Healthcare, Inc., Warren, NJ, United States; ^17^ Department of Health Systems & Implementation Science, Virginia Tech Carilion School of Medicine, Division of Allergy-Immunology Carilion Clinic, Roanoke, VA, United States

**Keywords:** APDS, EHR query, AI, inborn errors of immunity, diagnostic delay, IEI diagnosis

## Abstract

**Introduction:**

Patients with activated PI3Kδ syndrome (APDS) may elude diagnoses for nearly a decade. Methods to hasten the identification of these patients, and other patients with inborn errors of immunity (IEIs), are needed. We sought to demonstrate that querying electronic health record (EHR) systems by aggregating disparate signs into a risk score can identify these patients.

**Methods:**

We developed a structured query language (SQL) script using literature-validated APDS-associated clinical concepts mapped to *ICD-10-CM* codes. We ran the query across EHRs from 7 large, US-based medical centers encompassing approximately 17 million patients. The query calculated an “APDS Score,” which stratified risk for APDS for all individuals in these systems. Scores for all known patients with APDS (n=46) were compared.

**Results:**

The query identified all but one known patient with APDS (98%; 45/46) as well as patients with other complex disease. Median score for all patients with APDS was 9 (IQR = 5.75; range 1-25). Sensitivity analysis suggested an optimal cutoff score of 7 (sensitivity = 0.70).

**Conclusion:**

Disease-specific queries are a relatively simple method to foster patient identification across the rare-disease spectrum. Such methods are even more important for disorders such as APDS where an approved, pathway-specific treatment is available in the US.

## Introduction

Activated PI3Kδ syndrome (APDS) is an inborn error of immunity (IEI) with a heterogenous clinical presentation with signs of both immune deficiency and immune dysregulation (1–3). Hallmarks of immune deficiency include recurrent sinopulmonary infections and susceptibility to herpesvirus viremia, while dysregulation may manifest as atopy, lymphadenopathy, organomegaly, and autoimmunity (2, 4, 5). The proximate cause of increased risk of lymphoma in APDS is debated, but both deficiency and dysregulation could be implicated (6).

The complex array of symptoms frequently drives a patient to seek care from several types of clinicians. Uncoordinated care, misdiagnoses, status as a rare disease, range of severity of symptoms, unavailability of genetic testing, and other factors may contribute to the 7-year median diagnostic delay (7, 8). APDS is a progressive illness; therefore, like many IEIs, this diagnostic odyssey may increase healthcare utilization, lead to permanent organ damage such as bronchiectasis, and result in other poor outcomes (4, 7, 9–11).

Organizations such as the National Organization for Rare Disorders and Jeffrey Modell Foundation have led robust educational initiatives focused on rare diseases and IEIs specifically (12–14). Despite these and other efforts, diagnostic rates of IEIs remain stable apart from extreme presentations in infants (11, 15). The growing fields of bioinformatics and artificial intelligence investigate and provide methods to search electronic health records (EHRs) to weave together the patient encounters distributed throughout a hospital system (16–18). We sought to demonstrate that querying EHR systems to aggregate the disparate signs into a risk score could help identify patients with APDS.

At study origination, APDS did not have its own *ICD-10-CM* code, though it has since been assigned a code (81.82). This will presumably raise awareness of the disease; however, to our knowledge, there has been no impact on patient identification to date. We surmise that undiagnosed patients with APDS could be more efficiently identified by EHR query methods. This may be especially useful in the adult population, who may more easily escape diagnoses of an IEI (11, 15). Firstly, genetic disease has the bias of assumed fulminant presentation while the patient is young. Secondly, smoldering presentations in IEIs are not well characterized. They may remain unremarkable, present differently from the pediatric population, or become urgent only in adulthood (19, 20). For example, lymphoma was reported as the first clinical sign of APDS in 20.5% of patients in one cohort (n=39) who had a primary diagnosis charted (7).

Importantly, this disease-specific EHR query method could be mapped to the other nearly 500 IEIs. Interested researchers could also apply structured (16) approaches to initially cull a cohort of high risk or leverage unstructured data approaches such as natural language processing (NLP) additively to this effort (17, 21).

## Methods

This study was approved by the Institutional Review Board (IRB; H-50212) of Texas Children’s Hospital (TCH), Houston, Texas, with reciprocal approvals by the IRBs at Children’s Hospital Los Angeles, Los Angeles, California, University of South Florida, Tampa, Florida, Mount Sinai School of Medicine, New York, New York, and Spectrum (now Corewell) Health, Grand Rapids, Michigan; as well as a standing IRB for informatics research at University of California Los Angeles, Los Angeles, California.

We developed a structured query language (SQL) script using literature-validated APDS-associated clinical features mapped to *ICD-10-CM* codes (https://www.icd10data.com/) to identify patients. The initial features used were based upon relevant APDS clinical signs described in the literature up until 2021 (**Supplementary File 1**).

The raw query was then refined by adding relative-risk weights to selected APDS clinical features as learned from clinical data and previously published (22) and noted in **Table 1**. Briefly, weights for each feature were mined from a cohort of verified IEI patients (n=1762) by comparing frequencies of clinical conditions to that of a medically complex and diverse set of controls (n=1698). Relative risk values for features relevant to APDS were calculated by normalizing counts per capita as described previously. The learned feature weights and strategy for this approach are noted in **Supplementary File 2**. APDS score was calculated by summing the weighted features detected for every member of a cohort. For example, if bronchiectasis (weight = 4) and pneumonia (weight = 2) were detected from an individual’s records, their APDS score would be 6. Initial query validation took place within the TCH enterprise data warehouse (EDW) (**Supplementary File 4**). Having found all known patients at this center, and a patient there who had known PTEN (phosphatase and tensin homolog) syndrome, we sought to validate it at other institutions with known patients with APDS. Ultimately, this disease-specific query extracts predefined codes on each member of a population, builds a cohort of patients with any one of the relevant codes, applies the specified weight to each code-based clinical concept (ie, only counted once if present numerous times) then aggregates and ranks population members by APDS score in descending fashion.

**Table 1:** Feature weights (22).

Feature name	Weight
Lymphoma	11
Splenomegaly/organomegaly	6
Herpes viral infections	6
Bronchiectasis	4
Lymphadenopathy	2
Pneumonia	2
Bronchitis	1
Enteropathy	1
Relevant labs and/or previous diagnoses (eg, elevated IgM, CVID with autoantibodies)	1
Nodular lymphoid hyperplasia	1
Otitis	1

CVID, common variable immunodeficiency; IgM, immunoglobulin M. For full list of corresponding ICD-10-CM codes, please see **Supplementary File 2**.

We further validated this query by running it through 6 additional large US-based hospitals with known patients with APDS. These patients have been diagnosed by genetic testing performed by the expert immunologists caring for and treating these patients. The query was run directly in the EHR systems at Mount Sinai, New York, NY; Helen DeVos, Spectrum Health, Grand Rapids, MI; Phoenix Children’s Hospital, Phoenix, AZ, Children’s Hospital of Los Angeles (CHLA), LA, CA, and University of South Florida (USF), Tampa, FL. At the University of California, Los Angeles (UCLA), Los Angeles, California, the query was run in a deidentified data warehouse (DDW). The query was initially written for Epic Systems Corporation software and table structure (ie, Clarity) (**Supplementary File 2**). The refined and weighted query was converted to a language compatible with Cerner by Precision Extract so it could be run at CHLA (**Supplementary File 3**).

**Figure 1:**
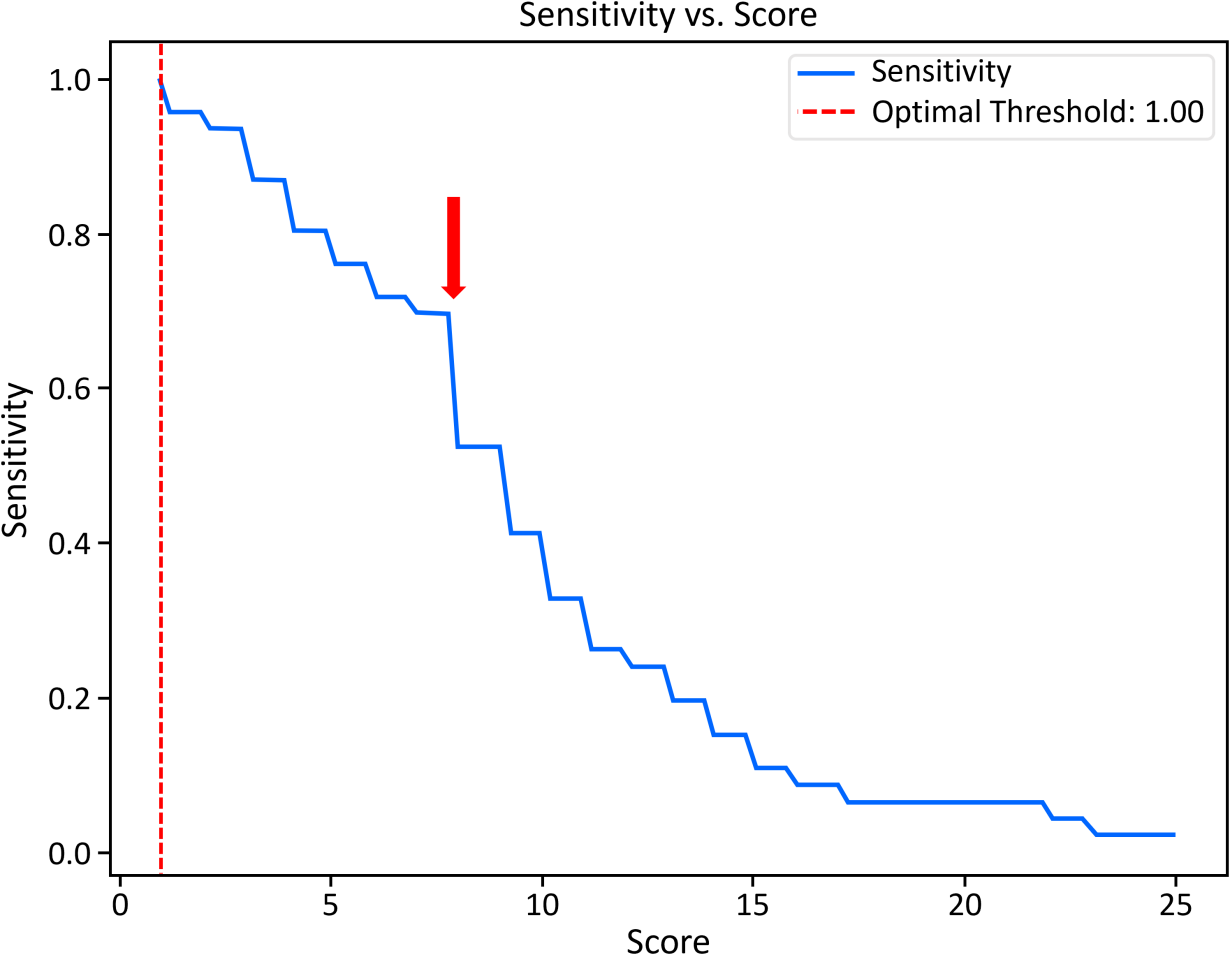
Sensitivity analysis for scores from all known patients with APDS. Balancing sensitivity with number of records captured, the arrow denotes an optimal cutoff score (7) and corresponding sensitivity of 0.70 calibrated from the centers evaluated here. Optimal threshold suggests a sensitivity of 100% and a score of 1, above which a very large number of subjects would be found effectively nullifying the need for our query.

Scores for all known patients with APDS were accumulated using all available data from each center. Median values and interquartile ranges were calculated with Python (3.12.4) and SciPy (1.11.1). A sensitivity analysis was conducted using the Python package NumPy (2.0.1) and plotted in Matplotlib (3.9.1). Optimal sensitivity was determined by plotting sensitivity vs score (**Figure 1**).

## Results

The query identified 98% (45/46) of patients with APDS across all centers (**Table 2**). One patient was not identified (ie, no scorable features in record); however, that individual is healthy and APDS was diagnosed pre-symptomatically via genetic testing prompted by family history (*PIK3R1*: c.343C>G p.Leu115Val). The median risk score for known patients with APDS across all institutions was 9, with a range from 1 to 25. The age range of known patients with APDS was 3 years to 69 years of age, with a median of 15 years of age. There did not appear to be a pattern of risk score vs age; however, this may be attributed to limitations of the EHR data. Sensitivity analysis suggested an optimal score cutoff of 7 (sensitivity = 70%). Across nearly 17 million individuals assessed, 41,041 (0.2%) had a score of ≥7 or 7741 patients per site (**Figure 1**). This represents an APDS high-risk population, which is reduced in number from the total population by more than 400-fold.

**Table 2:** Query results by institution.

Texas Children’s Hospital		Children’s Hospital Los Angeles		University of California, Los Angeles		Phoenix Children’s Hospital		Icahn School of Medicine at Mount Sinai		Helen DeVos Children’s Hospital and Corewell Health		University South Florida at Johns Hopkins All Children’s Hospital	
Starting Population ~2,100,000		Starting Population ~1,200,000		Starting Population ~4,300,000		Starting Population ~1,100,000		Starting Population 4,657,510		Starting Population 2,090,280		Starting Population 1,503,610	
Query Results													
APDS Score	Patient Age (y)	APDS Score	Patient Age (y)	APDS Score	Patient Age (y)	APDS Score	Patient Age (y)	APDS Score	Patient Age (y)	APDS Score	Patient Age (y)	APDS Score	Patient Age (y)
23	–	16	9	8	11	9	–	25	22	17	–	1	15
22	–	15	11	13	25	8	–	12	35	14	–	4	16
15	–	11	32	8	9	6	–	3	16	14	–	5	18
11	–	10	11	8	7	1	–			10	–	9	11
11	–	8	17	4	7	Null (not found)	–			9	–	10	39
10	–	8 (VUS)	8	4	21					8	–	13	13
9	–	6	25	3	69					7	–		
5	–	3	21	8	3								
				2	21								
				9	13								
**Median Score**	**Median Age**	**Median Score**	**Median Age**	**Median Score**	**Median Age**	**Median Score**	**Median Age**	**Median Score**	**Median Age**	**Median Score**	**Median Age**	**Median Score**	**Median Age**
**11**		**9**	**14**	**8**	**12**	**7**		**12**	**22**	**10**	**19**	**7**	**15.5**
Total records captured (based on lowest score)													
420,147		39,791		103,539		44,973		42,561		174,510		154,103	
Total records captured at or above median score % of records above median/total records | % of records above median/records captured													
Score of 11: **1262**		Score of 9: **130**		Score of 8: **9921**		Score of 7: **531**		Score of 12: **32**		Score of 10: **3415**		Score of 7: **18,764**	
0.06%	0.30%	0.01%	0.33%	0.23%	9.58%	0.05%	1.18%	0.0007%	0.08%	0.16%	1.96%	1.25%	12.18%
Records captured by score													
≥6: **12,184**		≥6: **1092**		≥6: **28,679**		≥6: **1020**		≥6:		≥6: **18,984**		≥6: **19,245**	
≥7: **7741**		≥7: **433**		≥7:		≥7: **531**		≥7:		≥7: **13,572**		≥7: **18,764**	
≥8: **6177**		≥8: **323**		≥8: **9921**		≥8: **473**		≥8:		≥8: **11,254**		≥8: **15,887**	
≥9: **3340**		≥9: **130**		≥9:		≥9: **107**		≥9:		≥9: **5842**		≥9: **10,630**	
≥10: **2172**		≥10: **86**		≥10:		≥10: **61**		≥10:		≥10: **3415**		≥10: **8809**	
≥11: **1262**		≥ 11: **60**		≥ 11:		≥ 11: **23**		≥ 11: **32**		≥ 11: **1748**		≥ 11: **7831**	
Maximum APDS score for all patients at this location													
**24**		**17**		**23**		**17**		**20**		**23**		**33**	

APDS, activated PI3Kδ syndrome; Y, years; VUS, variant of uncertain significance. Bolded values are for visual clarity and differentiation.

The results of the query at each institution are summarized in **Table 2**.

Some salient results not reflected in **Table 2** are as follows: UCLA, USF, and Mount Sinai each had patients with artificially low scores but for slightly differing reasons. At UCLA, the patients with scores of 2 and 3 had APDS features of bronchiectasis and splenomegaly listed in the notes. Further, one patient had marginal hyperplasia, but our query focused on nodular hyperplasia. If the query had also captured unstructured data and expanded the *ICD-10-CM* codes for hyperplasia, these patients would have had APDS risk scores of 7 and 12, respectively.

Similarly, when a patient is referred into the system at Mount Sinai, the patient’s history is primarily captured in the notes, and *ICD-10-CM* codes are rarely associated with these historical clinical features. Had there been codes in the structured data for the 3 patients’ complex histories, the scores would have been much higher. Further, these patients had cytopenias, which are a hallmark of APDS, but were not included in this script, and should be in future iterations (9).

At USF, the patient with a score of 1 was referred to the immunology department with a severe case of human papillomavirus (HPV). Our script focused on herpesviruses, a cardinal feature of APDS. However, HPV has also been reported. (4, 7, 23, 24). Up to 50% of patients with APDS have chronic viral infections with a wide range of presentations (25). This patient also had hypogammaglobulinemia and autism. These are common features of APDS and while not in the original query, we suggest adding them to future queries (2, 26).

Finally, variants of uncertain significance (VUSs) in either of the genes that cause APDS are of great interest. The query identified a patient at CHLA who has a VUS in an APDS gene, but two patients at USF who have VUSs in APDS genes were not identified.

## Discussion

We validated our query in seven large, US-based medical centers with 46 known patients with APDS. The query found all but one known patient with APDS (45/46), and that patient is asymptomatic. This relatively simple SQL script was a successful first effort and proof of concept for an EHR-based intervention to find patients with APDS, albeit with a suspected high false positive rate, that we draw attention to with refinement suggestions below. We suspect that this approach could find patients with other disorders of immune dysregulation that share clinical features and will probably be most useful in conditions with unique features or a complex and variable phenotype. Given that APDS and many IEIs are progressive, early diagnosis is critical to patient outcomes and to quality of life; thus, our aim was to create a process for reducing the diagnostic odyssey for patients with APDS. (4, 7, 9–11)

### Developing a diagnostic tool for a new disease

APDS was discovered in 2013, and though the impact of the genetic variants on the immune system was well described by both Angulo and Lucas, the understanding of clinical features in large cohorts continues to expand. (27–29) In 2017 and 2018, the ESID (European Society for Immunodeficiency) registry papers illustrated the main urgent immune features, but it was not until 2021 that atopy and asthma were well-established features of the disease. (2, 4, 9, 30) Thus, other important phenotypic features of APDS have only recently come to light. For example, neurological and behavioral concerns (26) and the specific timing of infections (31) are also recent attributes of the broader APDS phenotype. These findings, and those of other studies (32), emphasize the heterogeneous clinical presentation of APDS and suggest that we still have much to learn.

In newly discovered rare diseases without established natural histories, there may be considerable variation in which signs present, how those are captured in any EHR system, and how much of a patient’s history will be localized at the specialist’s hospital vs scattered among the records systems of the patient’s primary care physician, pulmonologist, gastroenterologist, hematologist, psychologist, and other clinicians. We have presented a script that captured the main features represented in the literature at that time, but we suggest updating this script before use.

### Refining this script for use in APDS

Though the script did find nearly all of the patients, it did so while capturing a significant number of presumed false-positive cases. The number of records returned for even the median APDS risk score at each hospital would not be immediately clinically actionable at most of the institutions. Though beyond the purview of our validation effort, many suggestions for refining the script exist. These are a few: running a targeted sensitivity analysis, then refining the script and rerunning it; adding additional *ICD-10-CM* codes, such as those for autism, hypogammaglobinemia, atopy, common variable immune deficiency or other immune disorders, which the patient may have been previously diagnosed with. Finally, the script could also be revised by adding a “number of encounters” or “events per year” feature, which would capture common signs (eg, atopy, otitis, specific infections) only if they appeared chronically across time or at an above-normal frequency per year, as recommended in the Jeffrey Modell Foundation 10 warning signs of immunodeficiency (33).

### Other IEIs could be identified

PTEN is a negative regulator of the PI3Kδ/ATP pathway; its deficiency can cause a hyperactivity of this pathway, leading to immunological manifestations similar to those observed in APDS (34). We were aware of a patient at TCH with PTEN deficiency and autism, and we intentionally confirmed that this patient record was captured by the script. Given the high record return, the script capture was unsurprising; however, we believe the script would find this record even after refinement. Similarly, it stands to reason that other patients who have IEIs of immune dysregulation (Category IV of the International Union of Immunological Societies classification system) could be identified with a refined version of this script (3).

### Navigating the use of artificial intelligence in a healthcare system

We have provided the open-source code (**Supplementary File 2**) to be refined accordingly and used for similar efforts at other institutions. There are many practical considerations to navigate. Efforts to codify the use of AI in large institutions are beginning to appear in the literature, and these roadmaps will be valuable (35, 36). Running a query like this can require coordination with several hospital stakeholders. In our experience, the IRB process varied institution to institution. The FDA guidance for AI tools is evolving (37). Presently, if the tools are considered to be diagnostic, the requirements related to IRB approval might be more significant than what we encountered for script validation. Further, one will need to identify the appropriate people in the information services (IS) department who can help ensure the query is compatible with each EHR, and who can run the query. We have included Epic- and Cerner-compatible scripts in the supplement, but there may be additional changes made based upon unique parameters in a hospital’s system. Finally, though deidentified data warehouses (copies of the hospital’s EHR system with the medical record numbers removed) are compelling systems for research and can expedite IRB approval, they create a nontrivial barrier to clinical implementation in queries like these. Without medical record numbers, it is not easy to refer the appropriate patients.

Our results displayed variability in part due to record management. This includes how each institution codes, refers, and records medical histories; some hospitals did not attach *ICD-10-CM* codes to any records transferred from outside systems, which meant the patients’ extensive histories were only captured in notes. The amount of care managed locally vs by the hospital specialist will also impact the results.

### Implementation of the results

The most important part of any patient identification effort is how to facilitate accurate diagnosis and access to appropriate care for newly discovered patients. We conducted a survey to this end. Expert considerations ranged from clinician availability to perform chart review, genetic testing and counseling to patient-centered identification and plan for unexpected financial burden. (38) Health systems will need to operationalize a workflow that is sensitive to existing and optimal clinical decision support processes for that organization. Critically, any healthcare analytics of this nature will require ongoing assessment and monitoring to ensure that its performance does not drift.

### Limitations

This was a proof-of-concept effort, and there are numerous limitations to this study. The evolving natural history of APDS is a significant limitation of the script. We included *ICD-10-CM* codes based on the literature at the time. We did not include cognition codes, which should be included. Also absent were autoimmune, atopy, cytopenias, additional viral infection codes, and some laboratory values. We suggest adding these to the script for further use.

Limitations of the data sets include patients who have few encounters with that system may be missed or have deceptively low scores. *ICD-10-CM* codes are useful structured data, but there is clinician-based variability in codes used. Some structured data may simply be absent or found in the unstructured patient notes. This last limitation can be corrected by using natural language processing models on top of the SQL query (17, 21).

Finally, this pilot study showcases the utility of a disease-specific query, which may not sufficiently detect other important IEIs or fully capture the phenotypic heterogeneity of APDS. Our experience, as described, offers a potential strategy to compress the diagnostic odyssey for patients with APDS but not patients with IEIs as a whole.

## Conclusion

EHR analytical approaches represent a strategy for reducing diagnostic delays among patients with APDS and other IEIs, but further methods to refine this search, and means to expeditiously evaluate new referrals, need to be devised. Our goal is to hasten diagnoses in these devastating diseases, and though AI is an excellent tool to do so, without the human intelligence to change large institutional practices, patients will languish undiagnosed.

## Data Availability

The data analyzed in this study is subject to the following licenses/restrictions: The data from the actual electronic health records cannot be supplied as it is not de-identified; however, the metadata is found in the article and the original source codes to generate data are found in the supplement.
