# Supplementary material for: Piloting an automated query and scoring system to facilitate APDS patient identification from health systems
Source: Front Immunol. 2025 Jan 21;15:1508780. doi: 10.3389/fimmu.2024.1508780 (PMC11790479; doi:10.3389/fimmu.2024.1508780)
Supplement: Supplementary file 1 [file DataSheet1.docx]

# **Electronic Health Record (EHR) instructions to create a patient query in the Epic^®^ EHR system**

[Electronic Health Record (EHR) instructions to create a patient query in the Epic^®^ EHR system 1](#_Toc63949739)

[1. Background, Instructions and Limitations 3](#_Toc63949740)

[3. SQL Statement 9](#_Toc63949741)

[4. Reporting Workbench or SlicerDicer 12](#_Toc63949742)

[5. Notes 14](#_Toc63949743)

#

# 1. Background, Instructions and Limitations

These instructions are created specifically for an EHR Data pull in the Epic EHR system and will not work for other EHR systems.

The process outlined below is variable, and not all steps will apply to every health system given the possible variation of data tables, naming conventions and database system set-up. Any steps or settings below that are not part of a health system’s standard process should be excluded or modified accordingly. Any questions should be directed to the appropriate service provider. The practice is solely responsible for implementing, testing, monitoring, and ongoing operation of any EHR tools.

The suggested criteria are listed below. Not every criteria may be routinely documented in the EHR and depending on the level of integration of the practice or health system, some criteria may be unavailable. Given this practice variation in documentation and coding, the list of criteria may be modified. A manual chart review of the resulting patient list is recommended.

The instructions detail the proposed steps to run the query in a Clarity environment and alternatively using Reporting Workbench. The final results can be exported to excel for further manipulation and to filter the results based on the number of results, weightings and other factors.

The SQL statement is a suggestion, there may be alternative methods to obtain the same results, therefore, it is recommended to align with the organization priorities and strategies. Local instances of the data tables and naming conventions may vary so adjust the SQL statement to reflect the local environment. As a result of the variability, the SQL statement may be altered to address any potential variation.

**Suggested Inclusion Criteria are in the table below:**

| Any patient with 2 or more of the following | Any patient with 2 or more of the following CATEGORIES | Incidence or qualifier |
| --- | --- | --- |
| Recurrent, severe sinopulmonary infections | | |
| - Pneumonia | **J15.0**, Pneumonia due to Klebsiella pneumoniae  **J15.7**, Pneumonia due to Mycoplasma pneumoniae  **J13**, Pneumonia due to Streptococcus pneumoniae **J15.3,** Bronchopneumonia due to group a streptococcus **J15.4**, pneumonia due to streptococcus, group B  **B01.2** Varicella pneumonia  **P23.9** Congenital pneumonia  [**J18.9**](https://www.icd10data.com/ICD10CM/Codes/J00-J99/J09-J18/J18-/J18.9), Pneumonia, unspecified organism  [A37.91](https://www.icd10data.com/ICD10CM/Codes/A00-B99/A30-A49/A37-/A37.91) , Pneumonia in pertussis  **J84.2**, Lymphoid interstitial pneumonia  [**J84.11**](https://www.icd10data.com/ICD10CM/Codes/J00-J99/J80-J84/J84-/J84.11) , Idiopathic interstitial pneumonia  [**P23.2**](https://www.icd10data.com/ICD10CM/Codes/P00-P96/P19-P29/P23-/P23.2),congenital  pneumonia   due to staphylococcus  [**J82.81**](https://www.icd10data.com/ICD10CM/Codes/J00-J99/J80-J84/J82-/J82.81), Chronic eosinophilic pneumonia  [**J16.8**](https://www.icd10data.com/ICD10CM/Codes/J00-J99/J09-J18/J16-/J16.8) , Pneumonia due to other specified infectious organisms, eg Fungal pneumonia  [**J84.111**](https://www.icd10data.com/ICD10CM/Codes/J00-J99/J80-J84/J84-/J84.111) , Idiopathic interstitial pneumonia, not otherwise specified  [**J18.1**](https://www.icd10data.com/ICD10CM/Codes/J00-J99/J09-J18/J18-/J18.1)**,** lobar pneumonia, unspecified organism  **P23, P23.6** Congenital pneumonia  [**J15.20**](https://www.icd10data.com/ICD10CM/Codes/J00-J99/J09-J18/J15-/J15.20)**,** Pneumonia due to staphylococcus, unspecified  **J12,**  Viral pneumonia, not elsewhere classified  **J85.1**, Abscess of lung with pneumonia  [J12.9](https://www.icd10data.com/ICD10CM/Codes/J00-J99/J09-J18/J12-/J12.9), Viral pneumonia, unspecified | ≥2 /year or in last 12 months |
| - Severe bronchitis and/or tonsillitis and/or sinusitis | J47.0, acute bronchitis with bronchiectasis  J44.0, acute bronchitis with chronic obstructive asthma  [**J20.9**](https://www.icd10data.com/ICD10CM/Codes/J00-J99/J20-J22/J20-/J20.9) , Acute bronchitis, unspecified Acute bronchitis; Acute exacerbation of chronic bronchitis; Chronic bronchitis with acute exacerbation  **J20.2,** Acute bronchitis due to streptococcus  [J20.1](https://www.icd10data.com/ICD10CM/Codes/J00-J99/J20-J22/J20-/J20.1)**,** Acute bronchitis due to Hemophilus influenzae  [**J20.0**](https://www.icd10data.com/ICD10CM/Codes/J00-J99/J20-J22/J20-/J20.0)**,** Acute bronchitis due to Mycoplasma pneumoniae [**J35.8**](https://www.icd10data.com/ICD10CM/Codes/J00-J99/J30-J39/J35-/J35.8)**,**  [**J03.01**](https://www.icd10data.com/ICD10CM/Codes/J00-J99/J00-J06/J03-/J03.01)  [**J20.8**](https://www.icd10data.com/ICD10CM/Codes/J00-J99/J20-J22/J20-/J20.8)**,** Acute bronchitis due to other specified organisms  Acute viral bronchitis  **J41.8,** Mixed simple and mucopurulent chronic bronchitis  [**J35.01**](https://www.icd10data.com/ICD10CM/Codes/J00-J99/J30-J39/J35-/J35.01) , chronic tonsillitis | ≥4 new infections per year or in last 12 months |
| - Severe otitis media   Especially if with encapsulated bacteria (eg, Haemophilus influenzae, Streptococcus pneumoniae) | [**H65.0**](https://www.icd10data.com/ICD10CM/Codes/H60-H95/H65-H75/H65-/H65.0)**,**Acute serous otitis media  **H66.93,** Otitis media, unspecified, bilateral  [**H65.3**](https://www.icd10data.com/ICD10CM/Codes/H60-H95/H65-H75/H65-/H65.3)**,** Chronic mucoid otitis media  [**H65.49**](https://www.icd10data.com/ICD10CM/Codes/H60-H95/H65-H75/H65-/H65.49)**,** Other chronic nonsuppurative otitis media  **H66,**Suppurative and unspecified otitis media | ≥4 new infections per year or in last 12 months |
| Severe or chronic Herpes infections   - Epstein Barr Virus (EBV) (chronic or with complications) - Cytomegalovirus (CMV)   (chronic or with complications)   - Zoster Virus (shingles) | **EBV mono**: B27.0, B27.09,  **CMV mono**:B27.1, B27.19  CMV pneumonia B25.0  Shingles B02  Herpesviral hepatitis B00.81  B25 Cytomegaloviral disease | B27.0 and B27.1 at 2x/year  Combination |
| Lymphadenopathy or chronic hepatomegaly or chronic splenomegaly  Lymphoproliferation | I88.1, Chronic lymphadenitis, except mesenteric  I88.0, mesenteric (acute) (chronic) lymphadenitis  R59, R59.0, R59.1, Enlarged lymph nodes  R59.9, enlarged lymph nodes, also lymphoid hyperplasia  R16.0, Hepatomegaly, not elsewhere classified  D73.2, Chronic congestive splenomegaly  D89.82, Autoimmune lymphoproliferative syndrome [ALPS]  J84.2, Lymphoid interstitial pneumonia | Suggest R59, R59.0, R59.1 Enlarged lymph nodes,  Listed twice |
| Any nodular mucosal lymphoid hyperplasia (NLH) | K63.89,  K31.7, Polyp of stomach and duodenum  R59.9, can be used for lymphoid hyperplasia – but it is not specific to mucosal |  |
| Autoimmune cytopenia, including but not limited to:  Coombs positive  hemolytic anemia, trilineage  cytopenia | D69.3, ITP  D59.10, Autoimmune hemolytic anemia, unspecified  D58.9, Hereditary hemolytic anemia, unspecified  D46.A, D46.B Refractory cytopenia with multilineage dysplasia |  |
| Enteropathy | K63.89, Other specified diseases of intestine  K63.9, Disease of intestine, unspecified  K58.9, IBS without diarrhea  K58.0, IBS w/ diarrhea  K52.89, Other specified noninfective gastroenteritis and colitis  K90 - Intestinal malabsorption  K52.9, Indeterminate colitis, Noninfective gastroenteritis and colitis, unspecified |  |
| Bronchiectasis | J47.9, J47.1, J47.0 |  |
| Lymphoma, including but not limited to:  large B-cell lymphoma  EBV positive lymphoma  nodular sclerosis  classical Hodgkin lymphoma  nodal marginal zone lymphoma,  lymphoplasmacytic lymphoma  Hodgkin-type lymphoproliferative  disorder | C86.2, C88.4, C81, C82, C83, C84, C85, C86, C88, C90, C94, C95, C96  D47.9 Other neoplasms of uncertain behavior of lymphoid, hematopoietic and related tissue |  |
| Onset of symptoms <12 years old |  |  |
| LABS | | |
| - Elevated IgM (immunoglobulin M) - Reduced CD3+ CD4+ T cells; - Elevated T follicular helper (Tfh) cells - Reduced naïve T cells | D80.5  D83.2 |  |

# 3. SQL Statement

DECLARE

@POST_START_DT VARCHAR(12),---

@POST_END_DT VARCHAR(12) ---

--@SERV_AREA_ID NUMERIC(18,0)

-- )

--AS

BEGIN

SET @POST_START_DT = '1/01/2021'

SET @POST_END_DT = '1/30/2021'

--SET @SERV_AREA_ID =

DECLARE

@END_INTERPRETED_DT datetime,

@START_INTERPRETED_DT datetime

SET @START_INTERPRETED_DT = CONVERT(DATETIME, dbo.UDF_RELATIVEDATE(@POST_START_DT), 101)

SET @END_INTERPRETED_DT = CONVERT(DATETIME, dbo.UDF_RELATIVEDATE(@POST_END_DT), 101)

SELECT DISTINCT

SERV_AREA_NAME,

LOC_NAME,

DEPARTMENT_NAME,

PROV_NAME,

LOC_NAME,

PAT_ID,

PAT_ENC_CSN_ID,

PAT_MRN_ID,

PAT_NAME,

REF_BILL_CODE AS ICD_CODE,

DX_NAME AS DIAGNOSIS_NAME,

CPT_CODE,

RCPT_MEM_SEX_C,

SEX,

SERVICE_DATE,

BIRTH_DATE,

CONTACT_DATE,

ENC_TYPE_C,

VISIT_TYPE,

APPT_STATUS_C,

APPT_STATUS,

ENC_CLOSED_YN,

APPT_MADE_DATE,

ORIG_APPT_MADE_DATE,

SCHED_APPT_DATE

FROM

(

SELECT DISTINCT

PAT_ENC.PAT_ENC_CSN_ID,

PATIENT.PAT_MRN_ID,

PATIENT.PAT_ID,

PATIENT.PAT_NAME,

CLARITY_EDG.DX_ID,

CLARITY_EDG.DX_NAME,

CLARITY_EDG.REF_BILL_CODE,

ARPB_TRANSACTIONS.CPT_CODE,

ZC_SEX.RCPT_MEM_SEX_C,

ZC_SEX.NAME AS SEX,

ARPB_TRANSACTIONS.SERVICE_DATE,

PATIENT.BIRTH_DATE,

PAT_ENC.CONTACT_DATE,

PAT_ENC.SERV_AREA_ID,

CLARITY_SA.SERV_AREA_NAME AS SERV_AREA_NAME,

CLARITY_DEP.REV_LOC_ID,

CLARITY_LOC.LOC_NAME AS LOC_NAME,

ZC_APPT_STATUS.APPT_STATUS_C,

ZC_APPT_STATUS.NAME AS APPT_STATUS,

PAT_ENC.ENC_TYPE_C,

ZC_DISP_ENC_TYPE.NAME AS VISIT_TYPE,

PAT_ENC.VISIT_PROV_ID,

CLARITY_SER.PROV_NAME AS PROV_NAME,

PAT_ENC.DEPARTMENT_ID,

CLARITY_DEP.DEPARTMENT_NAME AS DEPARTMENT_NAME,

PAT_ENC.ENC_CLOSED_YN,

PAT_ENC.APPT_MADE_DATE,

CONVERT(DATE, PAT_ENC.APPT_TIME) AS SCHED_APPT_DATE,

F_SCHED_APPT.APPT_MADE_DATE AS ORIG_APPT_MADE_DATE

--DATEDIFF(DAY,PAT_ENC.CONTACT_DATE,PAT_ENC.ENC_CLOSE_DATE) as LAG

FROM PAT_ENC

INNER JOIN ARPB_TRANSACTIONS ON PAT_ENC.PAT_ID = ARPB_TRANSACTIONS.PATIENT_ID

INNER JOIN CLARITY_EDG ON ARPB_TRANSACTIONS.PRIMARY_DX_ID = CLARITY_EDG.DX_ID

INNER JOIN PATIENT ON PAT_ENC.PAT_ID = PATIENT.PAT_ID

LEFT OUTER JOIN CLARITY_SER ON PAT_ENC.VISIT_PROV_ID = CLARITY_SER.PROV_ID

LEFT OUTER JOIN CLARITY_DEP ON PAT_ENC.DEPARTMENT_ID = CLARITY_DEP.DEPARTMENT_ID

LEFT OUTER JOIN CLARITY_SA ON PAT_ENC.SERV_AREA_ID = CLARITY_SA.SERV_AREA_ID

LEFT OUTER JOIN CLARITY_LOC ON CLARITY_DEP.REV_LOC_ID = CLARITY_LOC.LOC_ID

LEFT OUTER JOIN ZC_APPT_STATUS ON PAT_ENC.APPT_STATUS_C = ZC_APPT_STATUS.APPT_STATUS_C

LEFT OUTER JOIN F_SCHED_APPT ON PAT_ENC.PAT_ENC_CSN_ID = F_SCHED_APPT.PAT_ENC_CSN_ID

LEFT OUTER JOIN ZC_DISP_ENC_TYPE on PAT_ENC.ENC_TYPE_C = ZC_DISP_ENC_TYPE.DISP_ENC_TYPE_C

LEFT OUTER JOIN ZC_SEX ON PATIENT.SEX_C = ZC_SEX.RCPT_MEM_SEX_C

WHERE

PAT_ENC.CONTACT_DATE BETWEEN @START_INTERPRETED_DT and @END_INTERPRETED_DT AND

--(( @SERV_AREA_ID = 0 AND PAT_ENC.SERV_AREA_ID IN (7,22,26,27,28,29,44))

--OR @SERV_AREA_ID = PAT_ENC.SERV_AREA_ID)

------AND ENC_CLOSED_YN = 'Y' AND

ZC_APPT_STATUS.APPT_STATUS_C in ('1','2','6') --1= scheduled, 2=Completed, 6=Arrived,

AND CLARITY_EDG.REF_BILL_CODE IN ('A37.91',

'B00.81','B01.2','B02','B25','B25.0','B27.1',

'B27.19','C81','C82','C83','C84','C85','C86',

'C86.2','C88','C88.4','C90','C94','C95','C96',

'D46.A','D58.9','D59.10','D69.3','D69.42','D73.2',

'D80.5','D83.0','D83.1','D89.82','H65.0','H65.3',

'H65.49','H66','H66.93','I88.0','I88.1','J03.01',

'J12','J12.9','J13','J15.0','J15.20','J15.3','J15.4',

'J15.7','J16.8','J18.1','J18.9','J20.0','J20.1',

'J20.2','J20.8','J20.9','J35.01','J35.8','J41.8',

'J44.0','J47.0','J47.1','J47.9','J82.81','J84.11',

'J84.111','J84.2','J85.1','K52.89','K52.9','K58.0',

'K58.9','K63.89','K63.9','K90','P23','P23.2','P23.6',

'P23.9','R16.0','R59','R59.1','R59.9','R59.9')

--AND ORDER_MED.DESCRIPTION LIKE ('%METHOTREXATE%')--Rheumatoid arthritis, unspecified (CMS/HCC)

AND PAT_ENC.ENC_TYPE_C IN (50,101,125,212,243,244,251,257,293,294,304,308)

--AND PAT_ENC.PAT_ID = ''

--AND PATIENT.PAT_MRN_ID = ''

--AND PATIENT.BIRTH_DATE='DATEADD(yy,-12,CURRENT_TIMESTAMP) <= PATIENT.BIRTH_DATE

) RESULT

GROUP BY

SERV_AREA_NAME,

DEPARTMENT_NAME,

PROV_NAME,

LOC_NAME,

PAT_ID,

PAT_ENC_CSN_ID,

PAT_MRN_ID,

PAT_NAME,

DX_ID,

DX_NAME,

REF_BILL_CODE,

CPT_CODE,

RCPT_MEM_SEX_C,

SEX,

SERVICE_DATE,

BIRTH_DATE,

CONTACT_DATE,

VISIT_TYPE,

ENC_TYPE_C,

APPT_STATUS_C,

APPT_STATUS,

ENC_CLOSED_YN,

APPT_MADE_DATE,

ORIG_APPT_MADE_DATE,

SCHED_APPT_DATE

ORDER BY PAT_NAME

END

GO

# 4. Reporting Workbench or SlicerDicer

A Reporting workbench or SlicerDicer report could be created, by leveraging grouper records.

**Step 1: Create Diagnosis Grouper Records – Optional (only needs to be completed if no diagnosis grouper records are available)**

1. Access the Grouper Record Editor in Tools > Management Console

2. Select the Diagnosis master file and select the ICD-10 code set

3. Add all ICD-codes from each category of the inclusion criteria list to the diagnosis grouper and click Save

4. Repeat steps 1-3 until all diagnosis grouper records have been created

**Step 2 – Create the patient list**

**Option 1 – Reporting Workbench:**

1. Access Reporting Workbench (click the Epic logo > Reports > My Reports)
2. Navigate to the Library tab from the Reports menu
3. Enter “generic criteria” or “find patients” in the search field and click Search
4. Select the Find My Patients – Generic Criteria report and click New
5. The Report Settings field will display. Click the Criteria tab in the toolbar
6. Click Add and select a criterion. Enter “diagnosis” in the search field (Filter Criteria). Click Finish
7. Select the Diagnosis by Grouper criterion
8. Enter the Diagnosis Grouper records created in Step 1 in the search field and select all as a filter.
9. In the General tab, enter the desired Report Name (for example “*Pharming Inclusion Criteria Query”*) and a Description
10. Click Save and Run to create the patient list. The list will display all patients matching the criteria.
11. Export the results to excel. Set the desired columns to be included in the report.

**Option 2 – SlicerDicer:**

1. Access SlicerDicer (click the Epic logo > Reports > SlicerDicer)
2. Click New to start a new query
3. Click the drop-down arrow next to All Patients and select My Patients from the list
4. Click + Add Criteria. A new window will display
5. Select the All tab on the bottom of the SlicerDicer window to display all filters
6. Click the Diagnosis folder
7. Select the Diagnosis by Grouper filter from the available options
8. Enter the Diagnosis Grouper records created in Step 1 in the search field and select all as a filter.
9. Click Save As to save the query
10. Export the new report to Reporting Workbench
11. In the General tab, enter the desired Report Name (for example “*Pharming Inclusion Criteria Query”*) and a Description
12. Click Save and Run to create the patient list. The list will display all patients matching the criteria.
13. Export the results to excel. Set the desired columns to be included in the report.

# 5. Notes

- The Customers (i.e., physician, medical group, IDN) shall be solely responsible for implementation, testing, and monitoring of the instructions to ensure proper orientation in each Customer’s EHR system.
- Capabilities, functionality and set-up (customization) for each individual EHR system vary. Pharming shall not be responsible for revising the implementation instructions it provides to any Customer in the event that Customer modifies or change its software, or the configuration of its EHR system, after such time as the implementation instructions have been initially provided by Pharming.
- While Pharming tests its implementation instructions on multiple EHR systems, the instructions are not guaranteed to work for all available EHR systems and Pharming shall have no liability thereto.
- While EHRs may assist providers in identifying appropriate patients for consideration of assessment and treatment, the decision and action should ultimately be decided by a provider in consultation with the patient, after a review of the patient’s records to determine eligibility, and Pharming shall have no liability thereto.
- The instructions have not been designed to and are not tools and/or solutions for meeting Advancing Care Information and/or any other quality/accreditation requirement.
- All products are trademarks of their respective holders, all rights reserved. Reference to these products is not intended to imply affiliation with or sponsorship of Pharming and/or its affiliates.

Epic^®^ is a registered trademark of Epic Systems Corporation
