# Supplementary material for: Piloting an automated query and scoring system to facilitate APDS patient identification from health systems
Source: Front Immunol. 2025 Jan 21;15:1508780. doi: 10.3389/fimmu.2024.1508780 (PMC11790479; doi:10.3389/fimmu.2024.1508780)
Supplement: Supplementary file 3 [file DataSheet3.docx]

drop program apds_score_rpt:1 go

create program apds_score_rpt:1

free record pats

record pats(

1 cnt = i4

1 qual[*]

2 person_id = f8

2 name = c100

2 MRN = c20

2 feat_cnt = i2

2 apds_score = i4

2 bronchiectatis_flg = i2

2 bronchitis_flg = i2

2 entero_flg = i2

2 herpes_flg = i2

2 labs_flg = i2

2 lymphadem_flg = i2

2 lymphoma_flg = i2

2 nm_flg = i2

2 otitis_flg = i2

2 pneum_flg = i2

2 splen_flg = i2

)

declare i = i4 with noconstant(0)

;Set category weights as constants

declare bronchiectatis_wgt = i2 with constant(4)

declare bronchitis_wgt = i2 with constant(1)

declare entero_wgt = i2 with constant(1)

declare herpes_wgt = i2 with constant(6)

declare labs_wgt = i2 with constant(1)

declare lymphadem_wgt = i2 with constant(2)

declare lymphoma_wgt = i2 with constant(11)

declare nm_wgt = i2 with constant(1)

declare otitis_wgt = i2 with constant(1)

declare pneum_wgt = i2 with constant(2)

declare splen_wgt = i2 with constant(6)

declare out_file = vc with constant('$CCLUSERDIR:apds_score_rpt.csv')

select into 'nl:'

from person p

, person_alias pa

, diagnosis d

, nomenclature n

, code_value cv

where p.person_id = pa.person_id

and pa.person_alias_type_cd = 10 ;MRN code

and p.person_id = d.person_id

and d.diag_dt_tm >= cnvtdatetime('01-jan-2016')

and d.nomenclature_id = n.nomenclature_id

and (n.source_identifier in (

'J47.9','J47.1','J47.0', ;Bronchiectasis codes

'J47.0','J44.0','J20.9','J20.2','J20.1','J20.0','J35.8','J03.01','J20.8','J41.8','J35.01', ;Bronchitis codes

'K63.89','K63.9','K58.9','K58.0','K52.89','K90','K52.9', ;Enteropathy codes

'B27.0','B27.0','B27.09','B27.1','B27.19','B25.0','B02','B00.81','B25', ;Herpes codes

'D80.5','D83.2','D80.3','D72.810', ;Labs codes

'I88.1','I88.0','R59','R59.0','R59.1','R59.9','R16.0','D73.2','D89.82','J84.2', ;Lymphadenopathy codes

'C86.2','C88.4','C81','C82','C83','C84','C85','C86','C88','C90','C94','C95','C96', ;Lymphoma codes

'K63.89','K31.7','R59.9','D69.3','D59.10','D58.9','D46.A','D46.B', ;Nodular Mucosal codes

'H65.0','H66.93','H65.3','H65.49','H66', ;Otitis codes

'J15.0','J15.7','J13','J15.3','J15.4','B01.2','P23.9','J18.9','A37.91','J84.2',

'J84.11','P23.2','J82.81','J16.8','J84.111','J18.1','P23','P23.6','J15.20','J12','J85.1','J12.9','Z87.01', ;Pneumonia codes

'R16.1','D73.2','D73.81','R16.2','R16','R16.0','R16' ;Splenomegaly

)

or n.source_identifier = 'B27.0*' ) ;Other Herpes Codes

and n.source_vocabulary_cd = cv.code_value

and cv.code_set = 400

and cv.cdf_meaning = "ICD10*" ;ICD10 codes

and p.active_ind = 1

and pa.active_ind = 1

and d.active_ind = 1

and n.active_ind = 1

and cv.active_ind = 1

order by p.person_id, n.source_identifier

head p.person_id

if(mod(i,100) = 0)

stat = alterlist(pats->qual,i+100)

endif

i = i + 1

pats->qual[i].person_id = p.person_id

pats->qual[i].MRN = pa.alias

pats->qual[i].name = p.name_full_formatted

head n.source_identifier

if(n.source_identifier in ('J47.9','J47.1','J47.0'))

pats->qual[i].bronchiectatis_flg = 1

elseif(n.source_identifier in ('J47.0','J44.0','J20.9','J20.2','J20.1','J20.0','J35.8','J03.01','J20.8','J41.8','J35.01'))

pats->qual[i].bronchitis_flg = 1

elseif(n.source_identifier in ('K63.89','K63.9','K58.9','K58.0','K52.89','K90','K52.9'))

pats->qual[i].entero_flg = 1

elseif(n.source_identifier in ('B27.0','B27.0','B27.09','B27.1','B27.19','B25.0','B02','B00.81','B25')

or n.source_identifier = 'B27.0*')

pats->qual[i].herpes_flg = 1

elseif(n.source_identifier in ('D80.5','D83.2','D80.3','D72.810'))

pats->qual[i].labs_flg = 1

elseif(n.source_identifier in ('I88.1','I88.0','R59','R59.0','R59.1','R59.9','R16.0','D73.2','D89.82','J84.2'))

pats->qual[i].lymphadem_flg = 1

elseif(n.source_identifier in ('C86.2','C88.4','C81','C82','C83','C84','C85','C86','C88','C90','C94','C95','C96'))

pats->qual[i].lymphoma_flg = 1

elseif(n.source_identifier in ('K63.89','K31.7','R59.9','D69.3','D59.10','D58.9','D46.A','D46.B'))

pats->qual[i].nm_flg = 1

elseif(n.source_identifier in ('H65.0','H66.93','H65.3','H65.49','H66'))

pats->qual[i].otitis_flg = 1

elseif(n.source_identifier in ('J15.0','J15.7','J13','J15.3','J15.4','B01.2','P23.9','J18.9','A37.91','J84.2',

'J84.11','P23.2','J82.81','J16.8','J84.111','J18.1','P23','P23.6','J15.20','J12','J85.1','J12.9','Z87.01'))

pats->qual[i].pneum_flg = 1

elseif(n.source_identifier in ('R16.1','D73.2','D73.81','R16.2','R16','R16.0','R16'))

pats->qual[i].splen_flg = 1

endif

foot p.person_id

pats->qual[i].feat_cnt = pats->qual[i].bronchiectatis_flg +

pats->qual[i].bronchitis_flg +

pats->qual[i].entero_flg +

pats->qual[i].herpes_flg +

pats->qual[i].labs_flg +

pats->qual[i].lymphadem_flg +

pats->qual[i].lymphoma_flg +

pats->qual[i].nm_flg +

pats->qual[i].otitis_flg +

pats->qual[i].pneum_flg +

pats->qual[i].splen_flg

pats->qual[i].apds_score = (pats->qual[i].bronchiectatis_flg * bronchiectatis_wgt) +

(pats->qual[i].bronchitis_flg * bronchitis_wgt) +

(pats->qual[i].entero_flg * entero_wgt) +

(pats->qual[i].herpes_flg * herpes_wgt) +

(pats->qual[i].labs_flg * labs_wgt) +

(pats->qual[i].lymphadem_flg * lymphadem_wgt) +

(pats->qual[i].lymphoma_flg * lymphoma_wgt) +

(pats->qual[i].nm_flg * nm_wgt) +

(pats->qual[i].otitis_flg * otitis_wgt) +

(pats->qual[i].pneum_flg * pneum_wgt) +

(pats->qual[i].splen_flg * splen_wgt)

foot report

pats->cnt = i

stat = alterlist(pats->qual,i)

with nocounter

select into value(out_file)

patient_name = pats->qual[d1.seq].name

,pat_id = pats->qual[d1.seq].person_id

,MRN = pats->qual[d1.seq].MRN

,FeatureCOUNT = pats->qual[d1.seq].feat_cnt

,APDSScoreNBR = pats->qual[d1.seq].apds_score

,BronchiectasisFLAG = pats->qual[d1.seq].bronchiectatis_flg

,BronchiectasisWgt = bronchiectatis_wgt

,BronchitisFLAG = pats->qual[d1.seq].bronchitis_flg

,BronchitisWgt = bronchitis_wgt

,EnteropathyFLAG = pats->qual[d1.seq].entero_flg

,EnteropathyWgt = entero_wgt

,HerpesFLAG = pats->qual[d1.seq].herpes_flg

,HerpesWgt = herpes_wgt

,LabsFLAG = pats->qual[d1.seq].labs_flg

,LabsWgt = labs_wgt

,LymphadenopathyFLAG = pats->qual[d1.seq].lymphadem_flg

,LymphadenopathyWgt = lymphadem_wgt

,LymphomaFLAG = pats->qual[d1.seq].lymphoma_flg

,LymphomaWgt = lymphoma_wgt

,NodularMucosalFLAG = pats->qual[d1.seq].nm_flg

,NodularMucosalWgt = nm_wgt

,OtitisFLAG = pats->qual[d1.seq].otitis_flg

,OtitisWgt = otitis_wgt

,PneumoniaFLAG = pats->qual[d1.seq].pneum_flg

,PneumoniaWgt = pneum_wgt

,SplenomegalyFLAG = pats->qual[d1.seq].splen_flg

,SplenomegalyWgt = splen_wgt

from (dummyt d1 with seq = pats->cnt)

plan d1

where size(pats->qual,5) > 0

with format, nocounter, PCFORMAT('"',',',1,0), FORMAT = CRSTREAM

end

go
